# The Impact of Interleukin-17 Inhibitors on Major Adverse Cardiovascular Events in Psoriasis or Psoriatic Arthritis Patients Naive to Biologic Agents: A Systematic Review and Meta-Analysis of Randomized Controlled Trials

**DOI:** 10.7759/cureus.60980

**Published:** 2024-05-24

**Authors:** Ruoning Ni, Jiayi Zheng, Jimmy Varghese, Bharat Kumar

**Affiliations:** 1 Internal Medicine, University of Iowa Hospitals and Clinics, Iowa City, USA; 2 Internal Medicine, The Wright Center for Graduate Medical Education, Scranton, USA; 3 Rheumatology, Northwest Community Healthcare, Arlington Heights, USA; 4 Allergy and Immunology, University of Iowa, Iowa City, USA; 5 Rheumatology, University of Iowa, Iowa City, USA

**Keywords:** systematic review and meta analysis, il-17 inhibitor therapy, biological agents, psoriatic-arthritis, psoriasis treatment

## Abstract

The objective of this systematic review is to determine the effects of IL-17 inhibitors on major adverse cardiovascular events (MACEs) in patients with either psoriasis (PsO) or psoriatic arthritis (PsA). A systematic literature search in three databases (Medline, Embase, and the Cochrane Library for Randomized Controlled Trials) was conducted on December 7, 2022 for randomized controlled trials of patients with PsO/PsA treated with IL-17 inhibitors that reported confirmed MACEs. Two reviewers screened titles and abstracts and identified papers for full-text review. Exclusion criteria included trials that included the previous use of biological disease-modifying anti-rheumatic drugs. The Mantel-Haenszel random-effect method was utilized to calculate risk ratios and heterogeneity was measured by χ^2^ test and I^2^ statistics. Funnel plot analysis was undertaken to detect potential publication bias. Of the 919 references identified, nine RCT studies were included in the meta-analysis (n=2,096 patients). There was no statistically significant correlation between the use of IL-17 inhibitors and change in risk of MACEs (Risk Ratio 0.56; 95% CI 0.15 to 2.14; p* *= 0.40). Subgroup analysis of secukinumab or ixekizumab also did not demonstrate these changes. Additionally, there was no detectable dose-dependent effect of IL-17 inhibitors. In conclusion, IL-17 inhibitor use is not correlated with a change in MACE risk in patients with PsO/PsA who previously did not receive biologic disease-modifying anti-rheumatic drugs.

## Introduction and background

Psoriasis (PsO) and psoriatic arthritis (PsA) are increasingly being recognized as dermatological and articular manifestations of underlying systemic immune dysregulation that can affect multiple organ systems [1]. Patients with PsO and/or PsA are associated with a higher risk of atherosclerotic cardiovascular disease [2,3], including myocardial infarction (MI), closely related to the severity of PsO or PsA [4,5], as well as the duration of PsO or PsA [6].

Interleukin 17 (IL-17) is a key mediator of PsO and PsA, and Helper T-cell polarization towards Th17 plays an important role in the immunopathogenesis of disease. However, the role of IL-17, particularly IL-17A, in modulating cardiovascular disease is less well-known. In mouse models, the blockade of IL-17A reduces atherosclerotic plaque burden, inflammatory cell infiltration, cytokine/chemokine induction, and lesion development [7-11]. Further evidence indicates a role of IL-17A and Th17 cells in keratinocyte proliferation, angiogenesis, and intraplaque hemorrhage, which eventually lead to atherosclerotic diseases in human studies [12,13]. Patients with acute coronary syndrome also have higher levels of IL-17A acutely during episodes [14]. Higher serum levels of IL-17A are associated with recurrent I and may affect atherosclerotic plaque stability in the general population and inflammatory diseases [15,16]. Yet other lines of evidence suggest that IL-17 may play an anti-atherogenic role in stabilizing the atherosclerotic plaque and maintaining endothelium function [17].

This has important ramifications because several Il-17 inhibitors (IL-17i) have been investigated and have been approved by the Food and Drug Administration (FDA) for the treatment of PsA and PsO. These include a human monoclonal antibody specific for IL-17A (secukinumab), a humanized IgG4 against IL-17A (ixekizumab), and an antibody targeting the IL-17 receptor A (brodalumab). Additionally, a monoclonal antibody selectively neutralizing IL-17A and IL-17F (bimekizumab) has been submitted for FDA approval in December 2022.

The Evaluation of Cardiovascular Risk Markers in Psoriasis Patients Treated with Secukinumab (CARIMA) and Vascular Inflammation in Moderate-to-Severe Plaque Psoriasis (VIP-S) trials suggest a neutral to mild beneficial impact of secukinumab on endothelial dysfunction and aortic inflammation via serum biomarkers, flow-mediated dilation or FDG-PET/CT investigation [17,18]. However, these are surrogates for cardiovascular disease and may not necessarily reflect the totality of risk. This systematic review and meta-analysis seek to determine the associations between IL-17i and major adverse cardiovascular events (MACEs) in adult patients with PsO or PsA [19-22].

This article has been presented as a meeting abstract at the 2023 American College of Rheumatology Convergence meeting on November 13, 2023.

## Review

Materials and methods

The systematic review and meta-analysis were performed according to the Preferred Reporting Items for Systematic reviews and Meta-Analyses statement [23].

Search Strategy and Study Selection

A systematic search of the Medline, Embase, and Cochrane library databases was performed from inception through December 7, 2022. Search items included PsO, PsA, secukinumab, ixekizumab, brodalumab, bimekizumab, and randomized control trials (Table 1). Searches were limited to the English language. Two investigators (RN and RG) screened all titles and abstracts independently for inclusion. Discrepancies were resolved by third independent investigators (JZ and BK).

**Table 1:** Literature search strategy

Search number	Query	Filters	Results
1	((psoriasis) OR (psoriasis[MeSH Terms])) OR (psoriasis[Title/Abstract])		56,442
2	((psoriatic arthritis) OR (psoriatic arthritis[MeSH Terms])) OR (psoriatic arthritis[Title/Abstract])		12,029
3	(interleukin 17 inhibitors) OR (interleukin 17 inhibitors[Title/Abstract])		2,619
4	(secukinumab) OR (secukinumab[Title/Abstract]) OR (cosentyx) OR (AIN457)		1,466
5	(ixekizumab) OR (ixekizumab[Title/Abstract]) OR (taltz) OR LY2439821)		741
6	(brodalumab) OR (brodalumab[Title/Abstract]) OR (siliq) OR (KHK4827) OR (AMG827)		420
7	(bimekizumab) OR (bimekizumab[Title/Abstract]) OR (UCB4940)		61
8	#1 OR #2		58,777
9	#3 OR #4 OR #5 OR #6 OR #7		4,216
10	#8 AND #9		1,999
11	#8 AND #9	Randomized Controlled Trial	228

Inclusion and Exclusion Criteria

Studies were included only if they were randomized control trials (RCTs) that reported any adverse events in adult patients with PsO/PsA receiving IL-17i compared with placebo during the randomized controlled phase. Any dosage higher or more frequent than, not including 150mg every four weeks of secukinumab, 80mg every four weeks of ixekizumab, 140mg every two weeks of brodalumab, 160mg every four weeks of bimekizumab was regarded as high dose. Exclusion criteria included non-randomized design, non-comparative study, healthy volunteers, pediatric patients, only reported data on surrogate markers of atherosclerosis, not related to PsO/PsA or IL-17i, previous use of biologic disease-modifying antirheumatic drugs (DMARDs).

Data Extraction and Appraisal

Data were independently extracted by two reviewers (RN and RG) with a predefined data collection form that included authors, publication year, trial design, sample size, duration of follow-up, treatment regimens and all detailed MACEs. For extension RCTs in which treatment regimens were switched from placebo to an IL-17 inhibitor usually at week 12 or week 16, the incidence of MACEs was documented separately at switching point. For RCTs comparing two dosage arms, the respective number of MACEs for each dosage during the eligible period was also extracted to identify dose-associated cardiovascular effect. Selected studies were assessed for risks of bias using the Cochrane quality assessment tool for RCTs [24].

Outcome Measures

The primary outcome was the risk ratios (RR) of MACEs in patients receiving IL-17i compared to placebos. The major outcomes included MACEs. The MACEs are defined as all-cause mortality from cardiovascular events (CVEs), heart failure, non-fatal re-infarction, recurrent angina, re-hospitalizations for CVEs, repeat or unscheduled percutaneous coronary intervention, coronary artery bypass grafting and stroke 19,20. Two types of comparisons were made for the major outcomes: (1) all IL-17i which were classified into four subgroups: secukinumab, ixekizumab, brodalumab and bimekizumab versus placebo or DMARDs, all dosages were combined; (2) high dose versus low dose IL-17i. Sub-analysis of the RR of MACEs in patients with PsA receiving IL-17i compared to placebos was performed.

Data Synthesis and Statistical Analysis

Extracted data were prepared for meta-analysis using Review Manager software 5.4. Patient-years were calculated based on sample size and duration of follow up. RR were calculated by the Mantel-Haenszel random-effect method. The Mantel-Haenszel random-effects method assumes that different studies were estimating variant intervention effects and partly explains the heterogeneity between studies. Forest plots were constructed to summarize the RR estimates and their 95% CIs. Heterogeneity across studies was measured by χ2 test (p < 0.05 was regarded statistically significant) and I2 statistics (significant heterogeneity, I2 > 50%; insignificant heterogeneity, I2<40%). Funnel plot analysis was produced to detect potential publication bias.

Results

Study Selection

**Figure 1:**
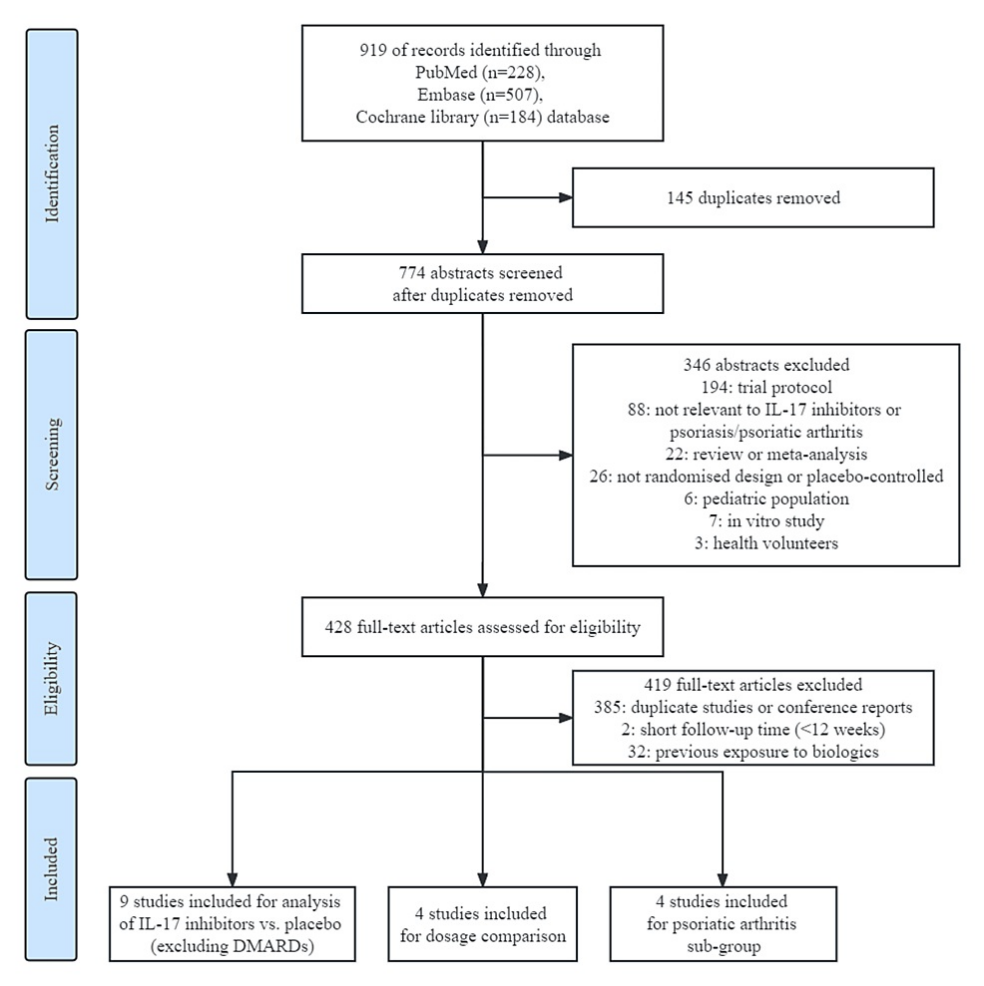
PRISMA flow diagram Abbreviations: IL-17 - Interleukin-17, DMARDs - Disease-Modifying Anti-Rheumatic Drugs

Nine hundred and nineteen references were identified through the literature search. Among them, 910 articles were excluded as they were duplicate studies, not randomized studies, not relevant to IL-17i or PsO/PsA, or previous exposure to biologic DMARDs (Figure 1). In total, nine RCT studies comprising 2,096 patients met the inclusion criteria (Figure 1) [25-33]. Among the trials included, nine studies were included for analysis of IL-17i versus placebo (totally 2,113 patient-years, 29 MACEs, see Table 2) [25-33] and four of nine studies for comparison of dosage (totally 1,615 patient-years, 24 MACEs) [27,30,32,33]. Four of nine studies were performed in patients with PsA (totally 1,728 patient-years, 27 MACEs) [30-33].

**Table 2:** Details of major adverse cardiovascular events

Trial	Authors	Publication year	Major Adverse Cardiovascular Events Details
IXORA-Q [25]	Guenther	2020	Peripheral ischemia and atrial fibrillation in the ixekizumab group
PRIME [26]	Sticherling	2017	No events
NCT01107457 [27]	Leonardi	2012	No events
NCT02634801 [28]	Reich	2020	No events
SCALP [29]	Bagel	2017	No events
SPIRIT-P1 [30]	Chandran	2020	A 59-year-old male with a history of dyslipidemia, diabetes mellitus, hypertension and previous transient ischemic attack experienced a fatal cerebrovascular accident while taking ixekizumab every 4 weeks. The event occurred 556 days after initiating ixekizumab every 4 weeks treatment.
ULTIMATE [31]	D’Agostino	2021	No events
MAXIMISE [32]	Baraliakos	2020	One death in the secukinumab 300 mg group was a case of ischemic cardiomyopathy in a 70-year-old male Caucasian patient with a known history of hypercholesterolemia and hypertension that happened on day 204; one myocardial infarction in the secukinumab 300 mg arm and one ischemic stroke in the secukinumab 150 mg arm.
CHOICE [33]	Nguyen	2022	One myocardial infarction in the secukinumab 150 mg group, and 1 ischemic stroke during placebo treatment. One death, due to cardiac arrest, was reported in a patient who received placebo prior to week 16.

The mean duration of follow up ranged from 12 to 156 weeks, with a mean of 46.2 weeks (standard deviation: 44.0 weeks). The disease activity in the IL-17i and placebo group was comparable. The incidence rates of MACEs in IL-17i and placebo group were 1.50 and 0.41 per 100 patient-years, respectively. Regarding the dosage comparison, the incidence rates of MACEs in high-dose and low-dose group were 1.55 and 1.42 per 100 patient-years, respectively.

Results of Meta-Analysis

**Figure 2:**
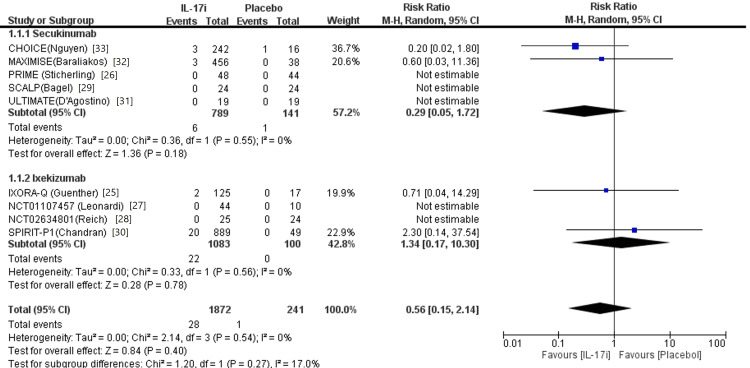
Forest plot of randomized control trials comparing IL-17 inhibitors to placebo in patients with both psoriasis and psoriatic arthritis Nine randomized controlled trials compared IL-17 inhibitors to placebo in patients having both psoriasis and psoriatic arthritis. Five randomized controlled trials compared secukinumab to placebo, while four randomized controlled trials compared ixekizumab to placebo.

**Figure 3:**
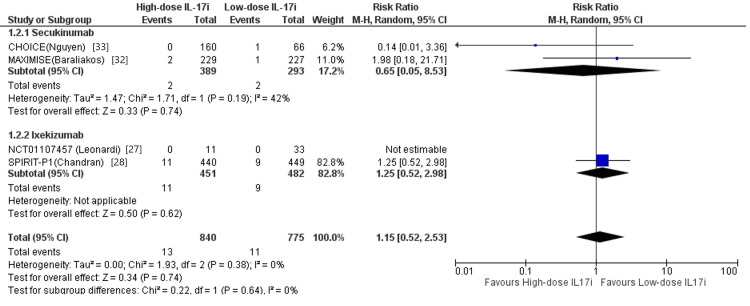
Forest plot of randomized control trials comparing IL-17 inhibitors to placebo in patients with only psoriatic arthritis

The analysis of nine trials indicated that use of IL-17i was not associated with an increased or reduced risk of MACEs in patients with PsO and/or PsA compared to placebo (RR 0.56, 95% CI 0.15 to 2.14, p = 0.40) in Figure 2. Regarding each IL-17 inhibitor, secukinumab (RR 0.29, 95% CI 0.05 to 0.72, p = 0.18) and ixekizumab (RR 1.34, 95% CI 0.17 to 10.30, p = 0.78) were not significantly associated with risk of MACEs. Patients in trials of brodalumab or bimekizumab have been exposed to biologic DMARDs to some extent, therefore, they were excluded from this review. No significant statistical differences were discovered in the sub-analysis in patients with PsA receiving IL-17i compared to placebo (RR 0.54, 95% CI 0.11 to 2.58, p = 0.44), neither in the subgroups (Figure 3).

At long-term follow-up beyond 52 weeks, the use of IL-17i still remained not significantly associated with risk of MACEs in patients with PsO and/or PsA (RR 0.56, 95% CI 0.15 to 2.14, p = 0.40). Dose comparisons for IL-17i were performed with no statistically significant difference in the risk of MACEs (RR 1.15, 95% CI 0.52 to 2.53, p = 0.74), neither in each sub-group in Figure 3. No significant heterogeneity was observed across all intragroup and intergroup analysis (χ2 =2.14, degree of freedom = 3, P = 0.54, I2 = 0).

Risk of Bias Assessment

**Figure 4:**
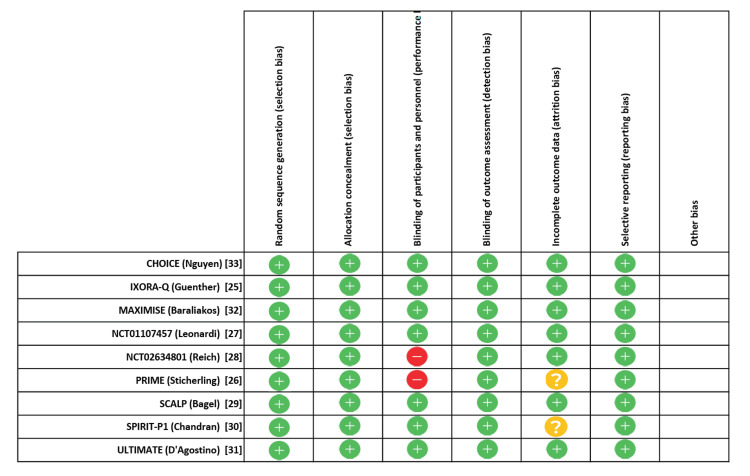
Risk of bias in included studies as assessed by the Cochrane collaboration’s “risk of bias” tool Among the nine trials included in this meta-analysis, there was overall a low risk of bias. Green plus signs reflect a low risk of bias, red minus signs reflects a high risk of bias, and yellow question marks signify unclear risk of bias.

**Figure 5:**
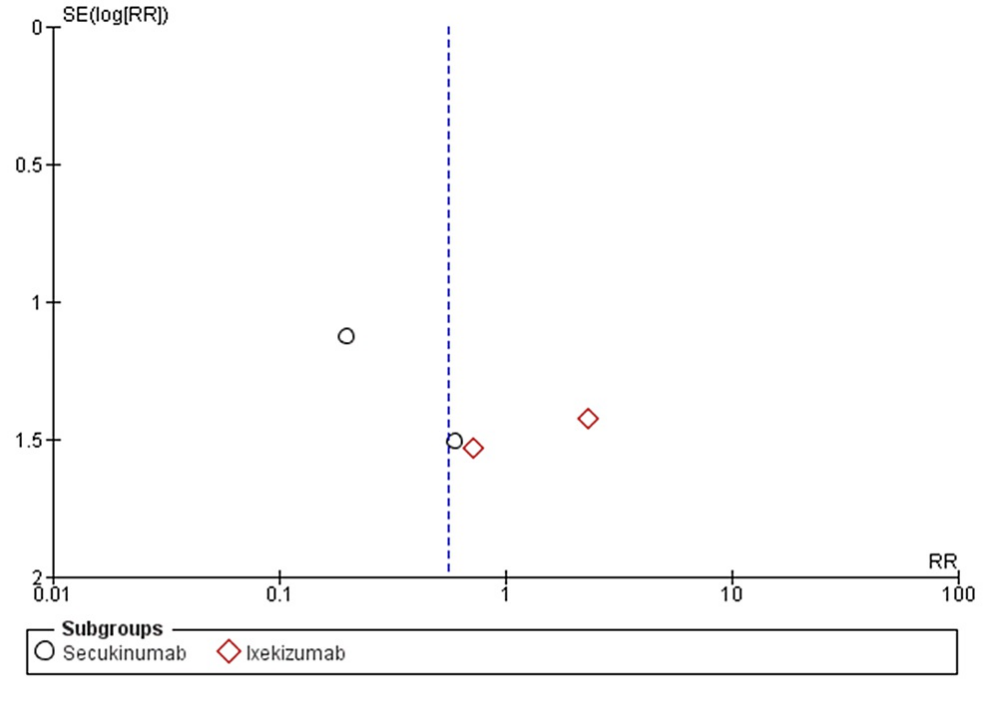
Funnel plot analysis for the secukinumab and ixekizumab subgroups using Mantel-Haenszel random-effects methods

Nine RCTs (100%) adequately reported generation of random sequence, nine RCTs (100%) adequately concealed allocation, seven RCTs (77.8%) blinded patients, investigators, nine RCTs (100%) blinded outcome assessors (Figure 4). Among nine RCTs, patients’ characteristics in all intervention groups were well balanced. For the Mantel-Haenszel random-effect methods, funnel plot analysis showed no evidence of publication bias in all comparisons (Figure 5).

Discussion

This meta-analysis did not reveal any statistically significant changes in the long-term risk for MACEs in PsO or PsA treated with IL-17i. Additionally, the dose-dependent effect of IL-17i on the risk of MACEs was undetectable. To our knowledge, this is the first meta-analysis determining the long-term impact of IL-17i on MACEs in patients with PsO or PsA.

This is significant in light of the strong evidence linking cardiovascular disease with PsO/PsA [4,5]. Multiple lines of evidence implicate IL-17A as a contributor to the pathogenesis of cardiovascular disease. Indeed, increased levels of IL-17A are detected in patients with acute coronary syndrome [14]. IL-17A also acts synergistically with interferon-γ to induce vascular smooth muscle cell inflammation [34]. IL-17A also activates human aortic endothelial cells, enhances human monocyte adhesion and up-regulates down streaming inflammatory cytokine pathways [35]. Considering the potential role of IL-17A in atherosclerosis and even MI [35], it is understandable why IL-17 has been considered a promising target for both controlling PsO and PsA disease activity and reducing cardiovascular risks.

In PsO and PsA, IL-17 produced from T-cells plays a crucial role in the immunopathogenesis of systemic inflammation [36,37]. IL-17i have been shown to be highly effective in reducing disease activity of moderate-to-severe PsO [38] or PsA [39,40], by ameliorating chronic systemic inflammation. The long-term concerns regarding cardiovascular safety have been less well-established. Several IL-17 inhibitors, including secukinumab and ixekizumab, have been approved for the treatment of PsO and PsA, and brodalumab has been approved as a therapeutic choice for PsO. Recent trials have also demonstrated rapid response with bimekizumab therapy [41,42]. It was reported that secukinumab demonstrated relatively favorable cardiovascular safety in patients with PsO and PsA [43]. No statistically significant difference was identified regarding secukinumab, ixekizumab and brodalumab with CVEs [44], with MACEs [22] and secukinumab and ixekizumab with MACEs [19]. However, it should be noticed that the mean duration of follow-up was 12 to 16 weeks and a small number of CVEs or MACEs occurred in the above included trials.

In light of the crucial role of IL-17A in the formation and maintenance of atherosclerotic plaques [8-10,12], it would be expected that secukinumab and ixekizumab, which directly target IL-17A, would confer a protective effect on atherosclerotic plaque formation and subsequent MACEs [17]. However, based on our results, secukinumab or ixekizumab did not provide any protective effect to the patients with PsO or PsA from MACEs, relative to placebo. In fact, patients administered a lower dose of IL-17i were more likely to have a lower risk of MACEs compared to those administered higher doses.

There are several possible explanations for this unexpected finding. First, it should be emphasized that lipid metabolism and endothelial injury remain dominant factors in the pathogenesis of cardiovascular disease, along with confounding factors, such as comorbidities. Secondly, disease activity may confound the signal for a protective effect. In SPIRIT-P1, MAXIMISE and CHOICE trials extension treatment period, non-responders were switched to a higher dose of IL-17i [30,32,33]. More severe disease activity in these non-responders could be associated with cardiovascular risk in patients receiving high-dose IL-17i, particularly in those with more severe PsO [45].

Another intriguing finding is that, while not statistically significant, there was a decreased risk of MACEs in patients with PsA receiving IL-17i not seen in the PsO group. This may be explained by the observation that the severity of atherosclerosis is higher in patients with PsA than patients with PsO [46,47]. Additionally, cardiovascular comorbidities are higher in patients with PsA than PsO [48].

It is also important to note that we deliberately excluded trials in which patients were previously exposed to TNF inhibitors. It has been shown that TNF inhibitors, notably etanercept and adalimumab, are associated with a reduced risk of developing MI compared to topical treatments in patients with PsO [5,49], as well as to disease activity control [50]. To distinguish the cardiovascular effects of IL-17i in patients with PsO and/or PsA, instead of determining the associations between controlled disease activities from DMARDs and cardiovascular effects, we excluded the trials which included patients with previous biologic use or comparing to other biologic DMARDs. The use of methotrexate was not associated with lower atherosclerotic events [51]. Therefore, we did not exclude patients using methotrexate in our study.

There remain limitations in our meta-analysis. First, the relatively low incidence of MACEs that occurred in placebo-controlled or comparative-controlled phases of the studies and 12- to 16-week duration of the placebo-controlled phases may reduce the power of our meta-analysis to detect a change in risk of MACEs. Including extension phases of trials can introduce bias from the drop off certain populations. Secondly, there was no stratification of cardiovascular risk factors when patients were allocated to the study agents. Moreover, the reviewers were not blinded to authors, or journals when screening the studies to be included in the meta-analysis, which might cause potential sources of bias. Additionally, we were unable to assess unpublished trials in the grey literature.

## Conclusions

In conclusion, our meta-analysis suggests that IL-17i, especially secukinumab and ixekizumab, do not alter the risk of major adverse cardiovascular outcomes in patients with PsO and PsA. Furthermore, there is no statistically significant dose-dependent effect on the risk of MACEs in patients with PsO and PsA. The results of this meta-analysis raise interesting questions and potentially new approaches to inquiry, such as investigating the differential roles of IL-17 isoforms and receptors in modulating cardiovascular risk in PsA and PsO patients and the clinical impact of combining DMARD therapy for further MACE risk reduction. Nevertheless, continuous post-marketing surveillance data is still required to ascertain the effect of IL-17i on cardiovascular outcomes in patients with PsO and PsA.
